# Effects of Rootstocks on Cryotolerance and Overwintering Survivorship of Genic Male Sterile Lines in Upland Cotton (*Gossypium hirsutum* L.)

**DOI:** 10.1371/journal.pone.0063534

**Published:** 2013-05-07

**Authors:** Xin Zhang, Zhiyong Zhang, Qinglian Wang, Peng Chen, Guoping Chen, Ruiyang Zhou

**Affiliations:** 1 College of Agronomy, Guangxi University, Nanning, Guangxi, China; 2 Cotton Research Institute, Henan Institute of Science and Technology, Xinxiang, Henan, China; East Carolina University, United States of America

## Abstract

Grafting desirable scion on stress-tolerant rootstocks provides an opportunity to improve the cryotolerance of scion. Genic male sterile (GMS) lines of plant could be used as sterile line and maintainer in breeding, and they have the conspicuous characteristics that the fertility of which is easy to regain but hard to maintain by sexual reproduction. In order to maintain the fertility of GMS cotton by means of its perennial growth on the basis of frostless winters in Nanning, Guangxi autonomous region, GMS line A4 was grafted onto 7 cryotolerant rootstocks (F118, F697, F098, F112, F113, P098 and P113), and the cryotolerance and the overwintering survivorship of scions were investigated. In consequence, when compared with control (self-grafted A4), the relative conductivity of the grafted plants in shoot bark was reduced (8.80%), the content of soluble sugar, soluble protein and free proline were higher, 25.00, 1.55, 3.46%, respectively; the overwintering survival rate and the height of regeneration bud under field condition of grafted plants were higher, 10.44, 15.75%, respectively; the order of the grafted plants based on the average subordinate function value of overwintering survivorship was A4/F113>A4/F118>A4/F098>A4/F697>A4/F112>A4/P098>A4/P113>A4/A4(CK); the correlation analyses indicated that the physiological parameters of cryotolerance could be used for forecasting the overwintering survivorship, and the relative conductivity could be chosen as the first physiological parameter for forecasting cryotolerance or overwintering survivorship. The results indicate that the cryotolerance and the overwintering survivorship of GMS cotton could be improved by grafting, and F113 appeared to be a valuable rootstock.

## Introduction

Cotton provides the primary textile in the world. Heterosis has been observed in many crops, including cotton, and it could be expressed over mid parent [1] or over a check hybrid [2], [3] in cotton. Heterosis breeding is an important genetic tool to improve yield and enrich many other desirable quantitative and qualitative traits in crops [4]. India and China are leading the way in commercial use of cotton heterosis. In 2009, of the total 9.6 million hectares in India, hybrid cotton occupied 90% [5], as in Yangtze River valley cotton-growing region of China.

There are mainly two seed production systems of cotton hybridization in China [6]: one is making emasculation and pollination by hand, the other is utilizing the male sterile line, and the former account for about 90%. When compared with making emasculation and pollination by hand, utilizing male sterile line to produce hybrid seeds could economize the cost of producing hybrid seeds and improve the efficiency which may promote the commercial use of cotton hybrid F_1_. Furthermore, cytoplasmic male sterile (CMS) line is more difficultly matched with maintainer and restorer than genic male sterile (GMS) line, which is randomly prepared mating combinations. For example, due to GMS lines “Dong A” have some merits including stable sterility, restored easily, etc. which were widely used for producing cotton hybrid F_1_ seeds in Sichuan province of China. However, the fertile plants, which account for about 50% of the total, should be removed during the florescence. That would waste tremendous labor and reduce the yield of F_1_ hybrid seeds. Even the two-step propagation and utilization of GMS cotton for producing F_1_ hybrid seeds was complicated and maintaining the male sterility of line MB was difficult. In addition, the price of cotton F_1_ hybrid seed is expensive and the supply of F_1_ hybrid seed is unable to meet the demand at present. Thus, it is very important to develop new methods for reducing the cost of hybrid cotton seeds.

Since cotton is a perennial plant in tropical and partial subtropical regions where the average temperature in the coldest month is above 10°C, we can make use of vegetative propagation to breed the GMS plants and maintain it perennially to solve the difficulties in heterosis utilization of cotton. Previous research [7] indicated that cutting propagation and perennial cultivation of GMS cotton used for producing hybrid seeds were feasible in Nanning, Guangxi autonomous region of China, but it could not ensure that all the stecklings overwintered safely when the winter temperature was lower than normal year.

Grafting plant shoots onto rootstocks to reduce the effect of external stress on the scion to increase production in cotton [8], [9]. This strategy was also used to study the interaction of scion and rootstock [10], [11] and improve the recovery of seedling plants from *in vitro* culture in cotton [12], [13]. Many results showed that the cryotolerance of scion was improved after grafted onto higher cryotolerant rootstock in some plant species like vegetables and trees [14]–[16]. For the effects of rootstocks on growth and vigor, it has been shown that rootstocks influence on the cryotolerance of grafted plants varies depending on rootstock choice [17]. Researches on perennial cotton indicated that the perennial species hold higher anti-reversion force, especially better overwintering survivorship [18], but lower yield and longer growth period than annual cultivars [19]–[21].

A grafting experiment was thus conducted to study the effects of rootstocks on the cryotolerance and the overwintering survivorship of genic male sterile lines in cotton. Annual GMS line A4 was grafted onto 7 different perennial rootstocks (F118, F697, F098, F112, F113, P098 and P113). The physiological parameters in scion bark of the grafted plants were determined, and the overwintering survivorships were investigated in the open field for selecting the best rootstock and trying to find some good physiological parameters of cryotolerance which could be used for forecasting the overwintering survivorship. To overcome the deficiency of estimating the cryotolerance and the overwintering survivorship of plant by only a single physiological parameter, the subordinate function method using fuzzy-set theory [22] was used to synthesize various correlated traits for estimating comprehensively.

## Materials and Methods

### The geographical and climate condition for experiment site

The field trial was carried out in the experimental field of Guangxi University (22°56′N, 108°21′E), where the annual mean temperature of past years was 21.6°C, the average temperature of the hottest month (July) was 28.3°C, the average temperature of the coldest month (January) was 12.3°C, ≥10°C active accumulated temperature amount to 7370.5°C, and the average precipitation was 1300.6 mm. The soil of experimental plot was krasnozem and the preceding crop was ramie (*Bochmeria nivea* L. Gaud.).

### Plant materials

Seeds of 7 rootstocks (F118, F697, F098, F112, F113, P098 and P113) and 1 scion (GMS lines A4 in upland cotton, called "A4" for short hereinafter) were sowed on May 4^th^, 2008. Five rootstocks, F118, F697, F098, F112 and F113 were F_1_ hybrids between upland cotton and island cotton, and other two rootstocks, P098 and P113 were homozygous island cotton. The scion of all grafted plants was A4 and the self-grafted A4 was used as the control.

In 2008, extreme low-temperature and rainy disaster weather occurred over a ten-day period in the middle of January and another ten-day period in the middle of February in Nanning, and the overwintering survival rate of A4 and all the rootstock materials planted in the open field was 0 and 100%, respectively.

### Cleft grafting

On May 19^th^, 2008, the shoots of the scion were grafted onto rootstock seedlings during the third true leaf stage. The modified grafting method [23] was described briefly as follows: the rootstock seedlings with stems 0.3 to 0.5 cm in diameter were cut transversely 4 to 5 cm above the seed leaves. The scion seedlings with the diameter similar to that of the ready rootstocks with 2 to 3 leaves were cut 2 to 3 cm above the seed leaves transversely and 1 to 2 cm long bevels were cut at the opposite sides below the buds. Then, the stocks were cleft about 2 cm deep from the top and the scions were inserted into the cleft quickly. Finally, the grafted seedlings were wrapped with sticky paper tightly. After grafting, the grafted plants were put into a small plastic arched shed which would be overshadowed if the inner temperature was too high and the inner moisture should be elevated for about a week so as to survive easily.

### Field experiment design

On May 27^th^, 2008, the testing seedlings were transplanted to the field with 3 replications in a completely randomized block design. Each block with an area of 7.5 m^2^, plant spacing of 0.75×0.75 m and 20 plants of each block were planted in double ridge rows.

### Determination methods

Leaf branches which held the consistent maturity and thickness from 5 representative plants in each block were chosen and cut half on December 31^st^ in 2008 when the daily mean temperature was about 10°C. Then, one of each testing materials was treated with 0°C for 48 h, and the other was treated at 20°C as normal control. After 48 h, the shoot bark of all materials were peeled out and cut into chippings, which could be directly used for the determination of relative conductivity, soluble sugar and free proline.

One gram (1 g) shoot bark was thoroughly homogenized with a cold mortar and pestle in 10 ml phosphate buffer reagent (pH = 7.8). The homogenate was centrifuged at 10,000 g for 15 min. The clear supernatant liquid was used for determination of soluble protein.

#### (1) Determination of relative conductivity

Changes of cell membrane permeability were measured by the modified method of Dionisio-Sese and Tobita [24]. To determine electrolyte leakage, 0.5 g fresh samples were placed in beakers containing 20 ml deionized water. The beakers were placed in a vacuum pumping system for air exhaust about 15 min when the samples were all down into the water. After standing 20 min at the ordinary temperature and pressure, the initial electrical conductivity of the medium (EC_1_) was measured using a DDS-11AT digital conductivity meter (Leici Corporation, China). Then, the samples were autoclaved afterwards at 100°C for 15 min to completely kill the tissues and release all electrolytes. Samples were then cooled to ordinary temperature and the final electrical conductivity (EC_2_) was measured. The relative conductivity (RC) was expressed following the formula: RC = EC_1_/EC_2_×100.

#### (2) Colorimetric determination of soluble sugar content

The modified anthrone reagent [25] was made by dissolving 0.2 g anthrone into 100 ml 98.3% (w/v) cool sulfuric acid, and glucose was used to establish a standard curve. 0.5 g fresh sample and 25 ml deionized water in each test tube with plug was heated for 20 min in a boiling water bath. After filtrated, the mixture of 1.0 ml soluble sugar solution, 1.5 ml deionized water and 6.5 ml anthrone reagent was oscillated for 30 s, cooled down, and the OD value at 620 nm was detected.

#### (3) Colorimetric determination of soluble protein content

Protein content of each sample was determined according to Bradford [26] using the Coomassie Brilliant Blue G-250 method. A protein standard curve was established using dilution of bovine serum albumin (BSA) prepared in solubilization solution.

#### (4) Colorimetric determination of free proline content

The modified determination of free proline content was according to the method of Bates et al. [27]. Free proline content was extracted from 0.3 g of samples in 3 ml 3% (w/v) salicylsulfonic acid and estimated by using acid-ninhydrin reagent. The absorbance of fraction with toluene aspired from liquid phase was detected at 520 nm. Free proline content was determined using calibration curve.

#### (5) Investigated overwintering survivorship in the open field

In the spring of 2009, the overwintering survivor rate of each block and the height of the highest regeneration bud of each biennial plant in the open field were investigated.

### Composite estimate of cryotolerance and overwintering survivorship

Before comparing procedure, all the raw data for each trait of all the materials were converted using the subordination function method in fuzzy mathematics [28]. The subordinate function value (SFV) of each trait for cryotolerance and overwintering survivorship was calculated based on its correlation with cryotolerance or overwintering survivorship.

#### (1) Calculate the SFV of cryotolerance or overwintering survivorship

The cryotolerance or overwintering survivorship subordinate value (U) for each trait was calculated based on its correlation with cryotolerance or overwintering survivorship. If the measured trait is positively correlation with cryotolerance or overwintering survivorship will be:
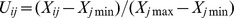



If the measured trait is adversely correlation with cryotolerance or overwintering survivorship will be:
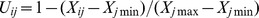



In the above-mentioned formula, 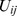 is the SFV of trait j in material i; 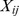 is an average value of trait j in material i, is the minimum average value of trait j in all materials; 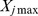 is maximum average value of trait j in all materials.

#### (2) Calculate the average SFV of cryotolerance or overwintering survivorship


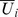
 represents the average SFV of cryotolerance or overwintering survivorship for material i, and 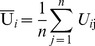
(n is the number of the measured traits).

## Results

### Changes of physiological parameters of cryotolerance in shoot bark of grafted plants after chilling treatment

**Figure 1:**
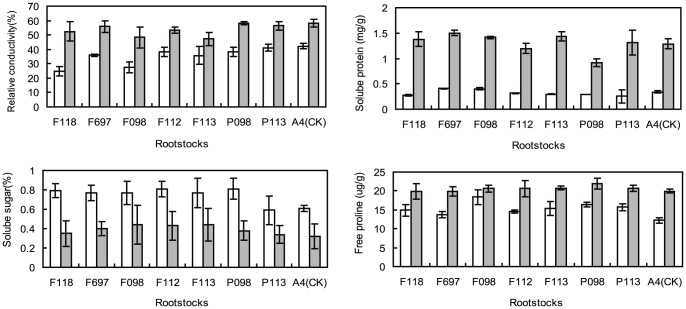
Changes of physiological parameters in the shoot bark of scion on different rootstocks. Relative conductivity, soluble sugar, soluble protein and free praline changed when exposed to 0°C (shade bar) and 20°C (white bar) for 48 h. Each value was the average of 3 repeats of separate shoots.

When exposed to 0°C for 48 h, the relative conductivity, content of soluble protein and free proline in all shoot barks were heightened greatly, but the soluble sugar content was greatly reduced, when compared with exposure to 20°C for 48 h (Figure 1). It indicated that the physiology metabolism was in the emergency for resisting chilling, and the cytomembrane was damaged under the low-temperature condition, and the leakage through the cytomembrane increased, but this damage showed some diversity among different grafted plants.

After chilling treatment, the relative conductivity of the grafted plants was significantly lower than that of the control, the contents of soluble sugar, soluble protein and free proline of the grafted plants were significantly higher than that of the control; and the relative conductivity of the grafted plant groups (F118, F697, F098, F112, F113) whose rootstocks were hybrids was lower, and the contents of free proline, soluble sugar and soluble protein of the latter were higher than that of the grafted plant groups (P098, P113) whose rootstocks were homozygous.

### Subordinate function values of cryotolerance in shoot bark of grafted plants after chilling treatment

After chilling treatment, it was shown that the relative conductivity of the grafted plants in shoot bark was lower (8.80%), and the content of soluble sugar, soluble protein and free proline which were higher (25.00, 1.55, 14.51, 3.46%, respectively) than that of the control (Table 1). In addition, it is very difficult to estimate the cryotolerance or overwintering survivorship of different plants by only a single physiological parameter (Table 2). The average SFV of cryotolerance of all grafted plants exceeded that of the control (self-grafted A4). The cryotolerance of grafted plants “A4/F113” was best among them.

**Table 1:** The physiological parameters change rate of grafted A4 after chilling treatment.

Parameter	Relative conductivity (%)	Soluble sugar (%)	Soluble protein (mg/g)	Free praline (μg/g)
Mean of not-self grafted A4	53.08±4.15	0.40±0.04	1.31±0.20	20.63±0.72
Self-grafted A4(CK)	58.20±2.91	0.32±0.13	1.29±0.10	19.94±0.53
Change rate by not-self grafting	−8.80%	25.00%	1.55%	3.46%

**Table 2:** The SFV of cryotolerance of grafted plants after chilling treatment.

	Subordinate function values (SFV)				
Scion/rootstock	RC	SS	SP	Pro	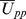
A4/F118	0.52	0.30	0.37	0.00	0.30
A4/F697	0.21	0.69	1.00	0.02	0.48
A4/F098	0.91	0.97	0.63	0.38	0.72
A4/F112	0.45	0.93	0.13	0.40	0.48
A4/F113	1.00	1.00	0.61	0.44	0.76
A4/P098	0.00	0.47	0.00	1.00	0.37
A4/P113	0.17	0.17	0.66	0.42	0.35
A4/A4(CK)	0.00	0.00	0.19	0.06	0.06

SFV means subordinate function value; 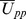 is the abbreviation of the average SFV of physiological parameters of cryotolerance.

### Analysis on overwintering survivorship of grafted plants in the open field

The overwintering survival rate and the height of the highest regeneration bud of each plant were investigated in the spring of 2009.

As shown in Table 3, the overwintering survival rate of all grafted groups under field condition were higher (mean, 10.44%) than that of the control (self-grafted A4), and grafted plants “A4/F113” was best among them (reached 100%); the height of the highest regeneration bud of all grafted groups were higher (mean, 15.75%) than that of the control, except grafted plants “A4/P098”. The average SFV for the overwintering survival rate of all grafted plants groups under field condition were higher than that of the control (self-grafted A4), the order of the grafted plants based on the average SFV of overwintering survivorship was A4/F113>A4/F118>A4/F098>A4/F697>A4/F112>A4/P098>A4/P113>A4/A4(CK).

**Table 3:** Analysis on overwintering survivorship of grafted plants in the open field.

	Overwintering survival rate		Height of regeneration bud			
Scion/rootstock	ADV (%)	SFV	ADV (cm)	SFV	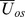	Order
A4/F118	96.67±3.33	0.75	61.72±2.71	0.91	0.83	2
A4/F697	96.67±3.33	0.75	59.86±6.06	0.77	0.76	4
A4/F098	96.67±3.33	0.75	60.82±8.36	0.84	0.80	3
A4/F112	93.33±3.33	0.50	55.13±2.59	0.41	0.46	5
A4/F113	100.00±0.00	1.00	62.88±8.71	1.00	1.00	1
A4/P098	96.67±3.33	0.75	49.73±2.06	0.00	0.38	6
A4/P113	90.00±5.78	0.25	55.50±4.30	0.44	0.34	7
A4/A4(CK)	86.67±8.82	0.00	50.07±5.62	0.03	0.01	8

SFV means subordinate function value; ADV means actual determination value; 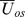 is the abbreviation of the average SFV of overwintering survivorship.

### Correlation analysis of the average SFV of cryotolerance and overwintering survivorship

Correlation analyses among the four measured physiological parameters, the average SFV of both cryotolerance and overwintering survivorship were shown in Table 4, the average SFV of physiological parameters was significantly correlated with the average SFV of overwintering survivorship, and the correlation coefficient was up to 0.79. While overwintering survivorship can be evaluated from observations in the field, the usefulness of such results is limited to the conditions, especially temperature, and there may be no difference among various materials when the winter temperature is too high or too low. Accordingly, laboratory methods of cryotolerance assessment have been developed and we could use the average SFV of cryotolerance to forecast the overwintering survivorship.

**Table 4:** Correlation analyses on the SFV of overwintering survivorship with physiological parameters of cryotolerance.

	Subordinate function values (SFV)				
Parameters	Relative conductivity	Soluble sugar	Soluble protein	Free proline	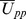
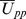	0.83**	0.91**	0.45	0.26	
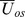	0.81*	0.69	0.54	−0.13	0.79*

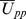
is the abbreviation of the average subordinate function values (SFV) of physiological parameters of cryotolerance; 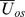 is the abbreviation of the average SFV of overwintering survivorship.*Correlation is significant at 95% probability.

In addition, among the four measured physiological parameters, only relative conductivity was significantly correlated with the average SFV of both cryotolerance and overwintering survivorship, and the coefficient correlations were up to 0.83 and 0.81, respectively, both of which reached the level of highly negative correlation. It indicated that the relative conductivity could be used as the first physiological parameter for forecasting cryotolerance or overwintering survivorship. Actually, relative conductivity was always chosen as the first physiological parameter when the cryotolerance in some plant species like field crops, vegetables and trees were investigated, since it was quick, easy, user independent, cheap and quantitative for identifying the cryotolerance [29]–[32].

**Figure 2:**
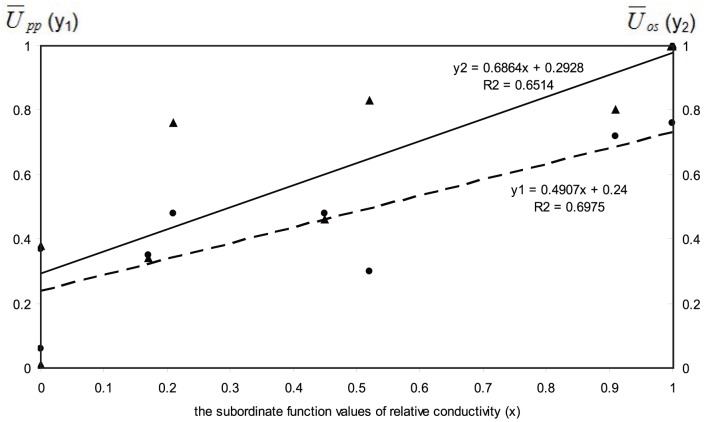
Forecasting the subordinate function values (SFV) of overwintering survivorship and physiological parameters of cryotolerance. Based on the SFV of relative conductivity (x) by linear regression, 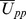
(y_1_) is the abbreviation of the average SFV of physiological parameters of cryotolerance and the relationship between it and relative conductivity is in the full line; 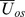
(y_2_) is the abbreviation of the average SFV of overwintering survivorship and the relationship between it and relative conductivity is in the dotted line.

For forecasting the SFV of overwintering survivorship and physiological parameters of cryotolerance based on the SFV of relative conductivity, the linear regression was used (Figure 2). The simple linear regression equation between 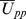
(y_1_) and the SFV of relative conductivity (x) is y_1_ = 0.4907x+0.24; and the simple linear regression equation between 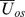
(y_2_) and the SFV of relative conductivity (x) is y_2_ = 0.6864x+0.2928.

## Discussion

Chilling stress occurs at temperatures lower than the plant's normal growth temperatures but not low enough to cause ice formation. Chilling is damaging for some plants primarily because of membrane leakiness caused by inability to increase membrane fluidity to accommodate the lower temperature [32]. It has long been established that injured cells are unable to maintain the chemical composition of their contents and may, therefore, release electrolytes through damaged membranes. The mechanism by which such events occur has still to be resolved; damage may be caused by reductions in cell volume following dehydration or by increasing concentrations of electrolytes [29]. The electrolyte leakage method of hypothermal injury assessment first described by Dexter et al. (1932) and the method of relative conductivity derived from it, Wilner [33] provides a less subjective method for scoring hypothermal injury and it has been widely used [30]. The accumulation of compatible solutes in the cytoplasm, such as soluble sugar and soluble protein, contribute to cryotolerance by reducing the rate and extent of cellular dehydration, by sequestering toxic ions, and/or by protecting macromolecules against dehydration-induced denaturation [34]. Free proline has been proposed to act as a hydroxyl radical and singlet oxygen scavenger [35], and to alleviate free-radical damage induced by chilling stress.

Grafting is regarded as a promising tool to broaden the temperature optimum of plant, the grafting process itself had no obvious effect on plant cryotolerance. The increased cryotolerance of grafted plant was due to “the use of cryotolerant rootstock”, plants grafted onto different rootstocks respond more or less differently to chilling [16]. This study concludes that grafting is an effective way to improve cryotolerance of cotton for the contents of soluble sugar, soluble protein and free proline in the bark tissue of the cryosensitive scion was increased after grafted onto the cryotolerant rootstocks which were selected from a chilling stress trial. These changes are correspondence with cryotolerance in plant tissue; however, it is hard to discern which is critical. Therefore, we supposed that acquisition of cryotolerance is a multifactor result of all these events and process [25]. That is why the subordinate function method in fuzzy mathematics was used to synthesize various physiological parameters correlated with cryotolerance for estimating accurately and comprehensively [28].

GMS lines of plant could be used as sterile line and maintainer in breeding, and they have the conspicuous characteristics of fertility is easy to regain but hard to maintain by sexual reproduction. In the present study, in order to maintain the fertility of GMS cotton by means of its perennial growth on the basis of frostless winters in Nanning, Guangxi autonomous region, GMS line A4 was grafted onto 7 different rootstocks (F118, F697, F098, F112, F113, P098 and P113), and the cryotolerance and overwintering survivorship of the grafted plants were investigated. In this study, the physiological parameters of cryotolerance could be used for forecasting the overwintering survivorship, and the relative conductivity may be the first physiological parameter for forecasting cryotolerance or overwintering survivorship. The results indicated that the cryotolerance and overwintering survivorship of GMS cotton could be improved by grafting, which is basically consistent with the researches in some plant species like cucumber [14], [15], tomato [16] and grape [36], and F113 appeared to be a valuable rootstock. The cryotolerance of grafted plant depends on not only the cryotolerance of rootstock, but also the compatibility between scion and rootstock. F113 was F_1_ hybrid of an upland cotton crossed with island cotton P113, the cryotolerance of grafted plant A4/F113 was better than A4/P113 which implied that maybe the heterosis of cryotolerance in F113 was positive and its compatibility with upland cotton A4 was better than P113.

In grafted plants, the rootstock absorbs water and nutrients, and composes hormone, proteins and metabolites, etc, which are transported to scion by graft union and affect the growth and development of scion. Grafting is also a well established technique for the growth and production of cotton, such as for controlling cotton *Verticillium wilt*, increasing yield in continuous cropping cotton field [9] and alleviating leaf senescence of early-senescent scion when grafted onto late-senescent rootstock [10], etc. The heterosis of hybrid is caused by the genetic composition of their parents which is not influenced by grafting, so there is no significant difference between hybrids produced from grafted GMS cotton and un-grafted GMS cotton.

GMS upland cotton propagated by grafting once overwintered safely could grow for producing hybrid seeds about 3 years in tropical and partial subtropical regions [37], [38], and it would omit the matched maintainer of the GMS line. Although it would need lots of labors to graft in the first year, but in the next two years, it would not need to plough, sow seeds, graft, etc. It would save lots of labors, seeds, fertilizer, etc, and it is conducive to the protection of the environment as well. However, it is still tedious in actual application, especially in the first year. In the future, it would not be necessary to propagate male sterile plants by grafting if the perennial GMS line of cotton with high overwintering survivorship, good synthetic properties, and high combining ability were bred. However, it is very difficult to be achieved for the perennial cottons have two fatal weaknesses: the growth period is too long and the yield is too low, even the perennial GMS lines were bred, if the sterile plants of them were not grafted, the fertile plants should be removed before producing hybrids in the first year. Consequently, grafting annual GMS scion onto perennial rootstock would be potential for perennial heterosis utilization in cotton, which could combine their merits and produce more F_1_ hybrid cotton seeds with high quality and inexpensive.
